# Premature mortality for patients after completely resected early adenocarcinoma of the esophagus or stomach

**DOI:** 10.1002/cam4.7223

**Published:** 2024-05-22

**Authors:** Elfriede Bollschweiler, Arnulf H. Hölscher, Sheraz R. Markar, Hakan Alakus, Uta Drebber, Stefan Paul Mönig, Patrick Sven Plum

**Affiliations:** ^1^ Medical Faculty University of Cologne Cologne Germany; ^2^ Center for Esophageal Diseases Elisabeth‐Krankenhaus Essen Essen Germany; ^3^ Surgical Interventional Trials Unit, Nuffield Department of Surgery University of Oxford Oxford UK; ^4^ Department of General, Visceral and Cancer Surgery University of Cologne Cologne Germany; ^5^ Institute of Pathology, University of Cologne Cologne Germany; ^6^ Department of Visceral Surgery Geneva University Hospitals Geneva Switzerland; ^7^ Department of Visceral, Transplantation, Thoracic and Vascular Surgery University Hospital of Leipzig Leipzig Germany

**Keywords:** adenocarcinoma, cancer survivors, esophageal neoplasm, esophagus mucosa, gastric mucosa, lymph node metastasis, stomach neoplasm, years of life lost

## Abstract

**Objective:**

To establish the life expectancy burden of esophago‐gastric cancer by analyzing years of life lost (YLL) for a Western patient population after treatment of early esophageal (EAC) or early gastric (GAC) adenocarcinoma.

**Background:**

For patients with early EAC or GAC, the short‐term prognosis after surgical resection is very good. Little data is available regarding long‐term prognosis when compared to the general population.

**Methods:**

Two hundred and fourteen patients with pT1 EAC (*n* = 112) or GAC (*n* = 102) were included in the study. Patients with EAC underwent transthoracic en‐bloc esophagectomy; those with GAC had total or subtotal gastrectomy with D2‐lymphadenectomy. Surviving patients had a median follow‐up of approximately 14 years. YLL was calculated using average life expectancy data from Germany.

**Results:**

Patients with EAC were younger (median age 61 years) than those with GAC (66 years) (*p* = 0.031). The male:female ratio was 10:1 for EAC and 3:2 for GAC (*p* < 0.001). Multivariate survival analysis showed the age of the patients ≥60 years and the existence of lymph node metastasis was associated with poor prognosis.

The median YLL for all patients who died over follow‐up was 8.0 years. For patients under 60 years, it was approximately 20 years, and for older patients, approximately 5 years (*p* < 0.001) without difference in tumor stage between these age cohorts. YLL did not differ for GAC vs. EAC.

**Conclusion:**

After surgical resection, the prognostic burden as measured by YLL is relevant for all patients with early esophageal and gastric adenocarcinomas and especially for younger patients. Reasons for YLL need further studies.

## Graphical abstract

Patients with adenocarcinoma of the gastroesophageal junction shows a reduced life expectancy compared to the standard population, although the tumor is completely resected. Possible reasons may be comorbidities, which are risk factors for this tumor entity.

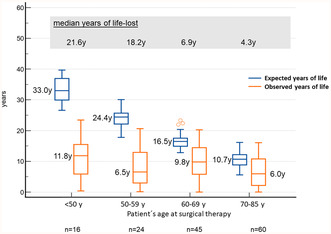

## 1 INTRODUCTION

Esophageal and gastric cancer are among the most biologically aggressive forms of solid‐organ malignancy.^1^ The geographical distribution and temporal trends of these cancers' incidence vary according to several factors including tumor morphology and primary localization.^2^ Although the histology of upper gastrointestinal tract adenocarcinoma (AC) is shared, many epidemiologic, biological, and histopathologic factors differ between the esophageal and gastric tumor entities.

The term “early” adenocarcinoma of the esophagus or stomach is used to describe tumors infiltrating the mucosa (T1a) and submucosa (T1b).^3^ Because lymphatic ducts are present in the lamina propria and muscularis mucosa deep to the basement membrane of the esophagus, T1a and T1b carcinomas are subdivided according to mucosal and submucosal thirds.^4^ Published results have indicated that lymph node metastasis (LNM) is very exceedingly rare in patients with carcinoma limited to the mucosa.

For patients with early AC of the esophagus or stomach limited to the mucosa, current German therapeutic guidelines recommend endoscopic or surgical resection; surgical resection is typically necessary for carcinomas infiltrating the submucosa.^5^, ^6^, ^7^ Current postoperative mortality is low, and five‐year survival rates are relatively high for patients with these circumscribed malignancies when compared to more common loco‐regional or metastatic esophageal or gastric cancer.^4^, ^8^, ^9^, ^10^

“Burden of disease” is a concept developed in the 1990s by the Harvard School of Public Health, the World Bank, and the World Health Organization (WHO) to characterize death and loss of health due to diseases.^11^ This concept is applied globally to map the health status of populations in a comprehensive and comparable manner according to a standardized concept.^12^

Patients undergoing complete resection of advanced tumors of the upper gastrointestinal tract continue with unfavorable prognoses because of microscopic metastasis. Thus, research and clinical studies have focused on incrementally improving the results of oncologic treatment. Thus, minimal data is currently available regarding the long‐term outcome of patients with the much more favorable diagnosis of early gastroesophageal AC with an established very low rate of cancer recurrence.^13^ Further existence of the risk factors, comorbidities, socio‐economic inequalities, or oncologic treatment may influence the prognosis of the patients.^14^ Whether life expectancy among successful treated patients with early gastric or esophageal cancer differs from the corresponding background population is unknown. More studies are needed evaluating risk factors for the development of those tumors, post‐therapeutic harms, or other patient‐related factors and its influence to reduced prognosis.

Premature mortality assessment offers one approach to evaluate the burden of this disease. The present study analyzed the years of life lost as a consequence of early death (YLL) in patients treated surgically for early gastric (GAC) or esophageal adenocarcinoma (EAC) by comparing the number of documented survival years with the average life expectancy of equivalent persons within Germany.

## 2 PATIENTS AND METHODS

### 2.1 Patients

Between 1996 and 2010, 530 patients diagnosed with EAC and 378 with GAC underwent surgical resection at the Department of Surgery of the University of Cologne. Histopathological analysis identified pT1 tumors in 112 patients with EAC and in 102 patients with GAC following primary surgery, excluding patients who had undergone neoadjuvant chemotherapy or radiochemotherapy (ypT1). These 214 patients with pT1 carcinoma met the inclusion criteria and were included in the present study.

### 2.2 Diagnosis

Esophageal adenocarcinomas (EAC) were classified if the center of the tumor was located at least 1 cm above the anatomic gastric cardia according to preoperative endoscopy, computed tomography scan, and postoperative examination of the resected specimen.^15^ Adenocarcinomas of the gastroesophageal junction (Siewert types II and III) were classified as GAC. Routine preoperative staging also included computed tomography scans and endoscopic ultrasound in all cases of early‐stage cancer. Preoperative risk analysis for evaluation of the organ function was performed.^16^

### 2.3 Surgery

In the beginning of the series in 1996, the endoscopic resection was not yet an established procedure. Therefore, the patients with cT1 esophageal carcinoma had esophagectomy. Later patients with mucosal carcinomas were increasingly treated with endoscopic mucosectomy, and those with submucosal carcinomas or recurrences after mucosal resection with esophagectomy. The treatment of choice for EAC was subtotal en bloc esophagectomy and gastric pull‐up with high intrathoracic esophago‐gastrostomy using laparoscopic gastric mobilization since 2003 and a right transthoracic approach including two‐field lymphadenectomy of the mediastinal and abdominal nodes.^17^ Some patients with adenocarcinomas type II, short esophagus, substantial hiatal hernia, or suspicious mediastinal lymph nodes also had transthoracic esophagectomy. Seven patients with EAC underwent transhiatal radical subtotal esophagectomy and cervical esophagogastrostomy because of distal localization and poor functional status.^16^ For patients with GAC, 85 total gastrectomies and 17 subtotal gastric resections were performed. Total gastrectomy was combined with transhiatal distal esophageal resection for 14 cases of types II and III AC of the gastroesophageal junction. All patients undergoing total gastrectomy had D2‐lymphadenectomy. Patients undergoing subtotal gastric resection had carcinomas of the distal stomach and had D2 lymphadenectomy excluding the right and left cardial nodes (Station Nos. 1 and 2), according to the Japanese Research Society of Gastric Cancer.^18^ The postoperative treatment included intensive care of all patients mostly for 1–3 days, and the start of oral intake 5–7 days postoperatively.

The specimens were removed en bloc, including all regional lymph nodes. To ensure the integrity of the primary tumor, the lymph nodes were dissected partially in the operating room and partially by pathologists according to a standardized protocol. On average, 30.4 (min: 13 – max: 64) lymph nodes for EAC and 28.7 (min: 14 – max: 73) lymph nodes for GAC were removed during the surgical procedures (not significant).

The standard reconstruction for patients receiving transthoracic esophagectomy was gastric pull‐up and high intrathoracic esophagogastrostomy.^17^ Three patients undergoing transthoracic and all patients undergoing transhiatal esophagectomy received cervical esophagogastrostomy. Reconstruction after gastrectomy was performed with Roux‐en‐Y end‐to‐side esophagojejunostomy, either to the abdominal esophagus or transhiatal to the esophagus in the lower mediastinum.

### 2.4 Histopathology

Histopathologic examination of all resected specimens comprised a thorough evaluation of tumor stage, residual tumor (R) category, grading, and the number of resected and involved lymph nodes. These data were documented at the time of surgery based on the original histopathological evaluations recorded by the pathologist.

All primary carcinomas were completely embedded. The specimens were fixed in 5% formaldehyde and embedded in paraffin. After fixation, the length and height of the tumors were measured. The lymph nodes were counted, and the maximum diameter of each node was measured with a slide gauge. A series of sections from each node was selected and stained with hematoxylin and eosin as well as with periodic acid‐Schiff (PAS). All dissected lymph nodes were microscopically analyzed for metastatic disease.

Using the 7th edition of the Tumor, Node, Metastasis (TNM) classification of malignant tumors,^3^ T1 tumors were further sub‐classified as T1a (limited to the mucosa or muscularis mucosae) and T1b (submucosal). The lesions were then subdivided according to invasion into three equal layers of one third of the mucosa (m1, m2, and m3) or submucosa (sm1, sm2, and sm3 referring to the upper, middle, and lower thirds, respectively). The depth of infiltration was measured at the deepest point of penetration by the cancer cells within the corresponding layer.

The neoplastic lesions were classified and graded according to the WHO recommendations.^19^ These data were reevaluated for an independent pathologic review during two earlier studies and were available for the present study.^4^, ^20^

### 2.5 Follow‐up

None of the patients with LNM had postoperative chemotherapy and/or radiation. Patients were followed up according to a standardized protocol. For the first 2 years, clinical follow‐up was performed at the hospital every 3 months. Afterwards, evaluations were carried out annually. Surviving patients had a median follow‐up of about 14 years with a maximum of 25 years. After 5 years, the survival versus death status of the patients was documented.

### 2.6 Statistics

We used the data of a prospective institutional data registry beginning in 1996 according to a standardized protocol. The aim of this esophageal/gastric cancer registry was to evaluate prognosis according to clinical factors like demographics of the patients, tumor stages, therapeutic methods, and so on. Descriptive analysis included the frequency of nominal parameters, the median with lower (LQ) and upper (UQ) quartiles for numeric variables (ordinal or asymmetric distribution), and the mean for numeric variables with normal distribution. Univariate analysis was calculated for tables using Chi‐squared statistics or Fisher's exact test if necessary. The Mann–Whitney *U* test was used to compare continuous variables. Analyzing influence factors for death the logistic regression was performed. Significant differences between groups were defined with a *p* < 0.05.

Univariate survival analysis was conducted according to the Kaplan–Meier method, and survival curves were compared with the log rank test. Multivariate analysis was performed with the Cox‐regression method. Prognosis was analyzed including postoperative mortality (90‐day mortality).

### 2.7 Control group

The patients of the study group with early cancer and complete resection had the very best prerequisites for a curative approach, which means long‐term freedom of cancer. This encourages the comparison to the life expectancy of the age‐ and gender‐adapted normal population (control group).

To evaluate the number of years of life, a person loses as consequences of dying early (YLL), we documented the number of expected years of life (EYL) for each patient. The EYL calculation was based on data from the Federal Statistical Office of Germany (Destatis) GENESIS V3.1. 2020.^21^ The EYL was calculated for the date of surgical therapy according to the age and sex of each patient and then registered. We used the average life expectancy (period life table) by years, sex, and completed age based on the year 2020. Details of calculation of this estimation are described in GENESIS.^21^ For patients dying during the follow‐up period, YLL was calculated as the difference between the expected time of death and the observed survival (OYS): YLL = EYL–OYS. We used the results of Kaplan–Meier survival analysis to estimate the YLL for all patients and subgroups.

The statistics program MedCalc® Statistical Software version 20.218 (MedCalc Software Ltd, Ostend, Belgium; https://www.medcalc.org; 2023) was used for statistical analysis.

### 2.8 Ethical approval

Written informed consent for collection of patient data was obtained from all patients within this study prior to surgery. This retrospective study was conducted and registered according to the actual guidelines of the local Research Ethics Commission.

## 3 RESULTS

### 3.1 Demographics

The median age of the 214 included patients was 63.5 years (min: 18, max: 84 years). Females were significantly older with a median age of 69.0 years versus males with 62.4 years (*p* = 0.001). Table 1 presents the demographic factors of the 112 patients with EAC compared to the 102 with GAC.

**TABLE 1:** Demographics and histopathologic data of 214 patients with early adenocarcinoma of the upper gastrointestinal tract.

Factor	Total *N*–%	Gastric Cancer *N*–%	Esophageal Cancer *N*–%	Significance
All patients	214–100%	102–100%	112–100%	
Age (median)	63.5 years	65.6 years	61.3 years	*p* = 0.031
Age group				*p* = 0.038
<60 years	87–40%	34–33%	53–47%	
60–85 years	127–60%	68–67%	59–53%	
Sex				*p* < 0.001
Male	166–78%	64–63%	102–91%	
Female	48–22%	38–37%	10–9%	
pT‐category				*p* = 0.056
pT1a (mucosa)	90–42%	36–35%	54–48%	
pT1b (submucosa)	124–58%	66–65%	58–52%	
pT‐subgroups				
Mucosa	90–100%	36–100%	54–100%	*p* < 0.003
m1	22–25%	3–8%	19–35%	
m2	12–13%	3–8%	9–17%	
m3	56–62%	30–84%	26–48%	
Submucosa	124–100%	66–100%	58–100%	*p* = 0.607
sm1	46–37%	24–36%	22–38%	
sm2	35–28%	21–32%	14–24%	
sm3	43–35%	21–32%	22–38%	
pN‐category				*p* = 0.130
pN0	181–85%	82–80%	99–89%	
pN+	33–15%	20–20%	13–11%	
pN‐category				*p* = 0.145
pN0	181–85%	82–80%	99–89%	
pN1 (1–2 LNM)	21–10%	15–15%	6–5%	
pN2 (3–6 LNM)	10–4%	4–4%	6–5%	
pN3 (>6 LNM)	2–1%	1–1%	1–1%	
No. of resected LN				*p* = 0.824
Median (LQ_UQ)	27 (18–37)	24 (17–43)	27 (19–34)	
R0‐resection rate	100%	100%	100%	*p* = 1.000

Abbreviations: LNM, lymph node metastasis; LQ, lower quartile; UQ, upper quartile.

Preoperative risk analysis showed elevated Body Mass Index (BMI ≥25) in 37%, cardiac comorbidities in 44%, pulmonal comorbidities in 25%, and peripheral arterial risk in 37% of the study cohort. 27 (24%) of the 102 EAC patients had one or more attempted endoscopic resections of the tumor before surgical intervention.

### 3.2 Depth of tumor infiltration and lymphatic invasion

In the patients with early GAC (*n* = 102), 35% (*n* = 36) showed mucosal infiltration (pT1a) compared to 48% (*n* = 54) in those with early EAC (*n* = 112). The frequency of LNM did not significantly differ between gastric and esophageal cancer (Table 1). Of the 36 patients with mucosa‐limited GAC, 8% (*n* = 3) presented with lymph node metastases (LNM). In comparison, none of the patients with pT1a EAC had LNM (*p* = 0.060). In cases in which the primary tumor infiltrated the submucosal layer, LNM were present in 26% (*n* = 17) for GAC and in 22% (*n* = 13) for EAC (*p* = 0.665). The number of resected lymph nodes was not significantly different between the two types of tumor location (Table 1).

We compared younger (<60 years) and older (≥60 years) patients according to the tumor stages and found no significant differences. In addition, the pT‐ and pN‐categories were not different between males and females (Table 2).

**TABLE 2:** Comparison of tumor stages between males and females, for different age groups and between gastric (GAC) and esophageal (EAC) carcinoma of all patients.

Factor	All Patients *N* %	pT‐category				Sign. *p*	pN‐category				Sign. *p*
		pT1a		pT1b			pN0		pN+		
		*n*	%	*n*	%		*n*	%	*n*	%	
	214,100%	90	42%	124	58%	–	181	85%	33	15%	–
Sex						0.085					0.277
Male	166–78%	75	45%	91	55%		138	83%	28	17%	
Female	48–22%	15	31%	33	69%		43	90%	5	10%	
Age group						0.825					0.266
<50 years	35–16%	17	49%	18	52%		26	74%	9	26%	
50–59 years	52–24%	22	42%	30	58%	*p*‐trend	44	85%	8	15%	*p*‐trend
60–69 years	65–31%	27	42%	38	58%	0.374	58	89%	7	11%	0.164
70–85 years	62–29%	24	39%	38	62%		53	85%	9	15%	
Location						0.0564					0.106
GAC	102–48%	36	35%	66	65%		82	80%	20	20%	
EAC	112–52%	54	48%	58	52%		99	88%	13	12%	

In a subgroup analysis for patients with completely fulfilled preoperative risk‐analysis (*n* = 108), 53% patients younger than 50 years showed high BMI compared to all other age groups with about 35%. Our data showed significantly (*p* < 0.002) less often coronary risks in the younger (24%) versus older (56%) group. Other risk factors were not different between both groups.

### 3.3 Prognosis

The 90‐day postoperative mortality was 3.3%. The median survival for all patients was 12.3 (95%CI = 10.9–13.9) years. The 5‐year survival probability (SP) for early carcinoma of the upper gastrointestinal tract was 76.6% (Table 3).

**TABLE 3:** Univariate and multivariate analysis of reasons for dying during the first 5 years and of those patients who died later during the follow‐up after surgical procedure for 214 patients with adenocarcinoma of the upper gastrointestinal tract.

Factor	All Patients	Patients died during first 5 y		Multivariate Regression Analysis Death during first 5 y		Patients survived 5 years or more	Patients died after 5 y during follow‐up		Multivariate survival analysis	
	*N*–%	*N*–%	Sign. *p*	H.R.	Sign. *p*	*N*–%	*N*–%	Sign. *p*	H.R.	Sign. *p*
All patients	214–100%	50–23.4%		–		164–76.6%	95–58%	–	–	
Sex			0.938		0.399			<0.001		0.858
Male	166–78%	39–23.5%		1		127–76.5%	63–50%		1	
Female	48–22%	11–22.9%		0.67		37–77.1%	32–86%		1.05	
Age group				<0.001				<0.001		
<50 years	35–16%	4–11.4%		1		31–88.6%	12–39%		1	
50–59 years	52–24%	11–21.2%		2.94 0.115		41–78.8%	13–32%		0.85 0.689	
60–69 years	65–31%	9–13.8%		1.83 0.384		56–86.2%	36–64%		2.18 0.022	
70–85 years	62–29%	26–41.9%		9.59 <	0.001	36–58.1%	34–94%		5.97 < 0.001	
Location			0.421		0.763			<0.001		0.201
GAC	102–48%	27–26.5%		1		75–73.5%	60–80%		1	
EAC	112–52%	23–20.5%		0.89		89–76%	35–39%		0.73	
pT‐category			0.049		0.475			0.397		0.267
pT1a (mucosa)	90–42%	15–16.7%		1		75–81%	41–55%		1	
pT1b (submucosa)	124–58%	35–28.2%		1.33		89–71%	54–61%		0.78	
pN‐category			<0.001		<0.001			0.861		0.827
pN0	181–85%	33–18.2%		1		148–81%	85–57%		1	
pN+	33–15%	17–51.5%		5.69		16–45%	10–63%		0.92	

Abbreviations: H.R., Hazard Ratio; Sign., Significance; y, years.

Multivariate analysis of prognosis including the factors location (GAC vs. EAC), age groups (<60 vs. 60 years or older), sex (male vs. female), pT‐category (pT1a vs. pT1b), and pN‐category (pN0 vs. pN+) showed that patient age and pN+ significantly influenced the total prognosis (age <60 vs. ≥60y: H.R. = 2.58, 95%CI = 1.78–3.91, *p* < 0.0001; pN0 vs. pN+: H.R. = 1.73, 5%CI = 0.46–0.94, *p* = 0.024).

Analyzing prognostic factors associated with death during the first 5 years after surgery, the existence of lymph node metastasis was the most relevant factor with H.R. of 4.95, 95%CI = 2.06–11.88 for pN+ category compared to pN0 (Table 3). This was most marked in pN+ patients, with tumor infiltration to sm2/sm3, who had a substantially worse 5‐year overall survival than those with m3/sm1 (*p* = 0.013).

One hundred sixty four (77%) of all patients survived 5 years or more after surgical therapy for early carcinoma. During the further follow‐up period 95 patients died, with multivariate survival analysis showed increasing age, as the most relevant factor for dying after 5 years (Table 3).

### 3.4 Years of life lost (YLL)

**FIGURE 1:**
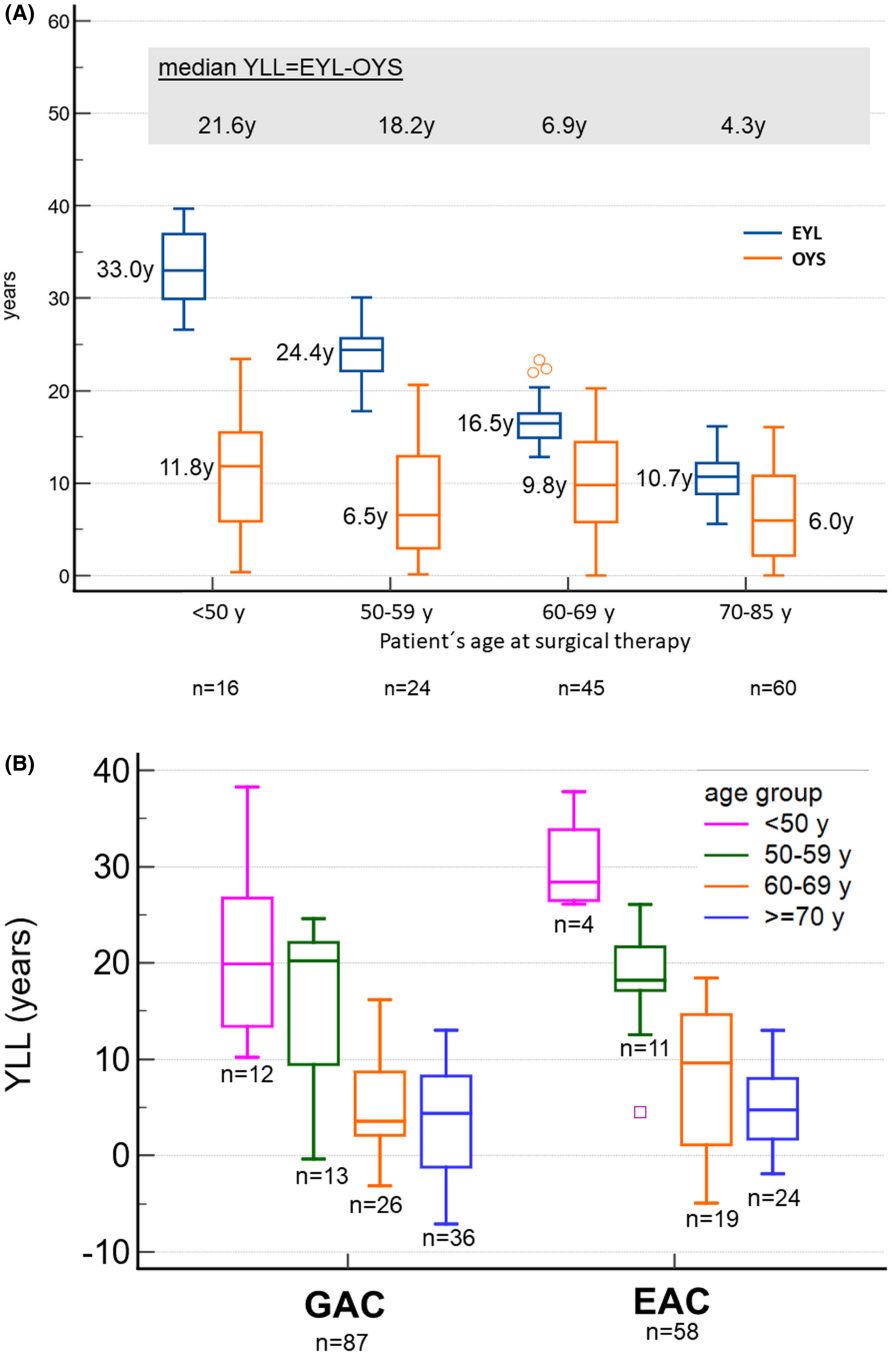
(A) Comparison of expected years of life (EYL) and years of observed survival (OYS) for 145 patients with early adenocarcinoma of the upper gastrointestinal tract who died over the follow‐up period. (B) Years of life lost (YLL) for different age groups of 145 patients who died over the follow‐up period comparing patients with early gastric and early esophageal cancer. EYL, expected years of life adjusted for gender and age at time of surgical therapy, OYS, observed survival in years after time of surgical therapy.

Overall, 145 patients (68%) of the study cohort died during the follow‐up period. For each patient of this subgroup, we calculated the YLL as the number of life years lost as a consequence of dying earlier compared to the life expectancy of the normal population. The median EYL for this group was 15.6 (95%CI = 13.7–16.5) years, and the median observed years of survival OYS was 7.8 (95%CI = 6.3–9.5) years. The median YLL for this group was 8.0 (95%CI = 6.0–9.1) years. Between the different age groups, the median OYS varied; the median OYS was 11.8 years for patients under 50 years and 6.0 years for patients 70 years and older (Table 4 and Figure 1). Younger patients in this group had more often LNM compared to the older patients (*p*‐trend = 0.013). The tumor stages according to the demographic factors for patients who died during the follow‐up are presented in Table 5.

**TABLE 4:** Univariate analysis of years of life lost (YLL) of 145 patients with early adenocarcinoma of the upper gastrointestinal tract who died during the follow‐up period.

Factor	Patients			Patients died during follow‐up			YLL median	95% CI	Significance *p*
	Study *N*	Died N	%	*N*	EYL median	OYS median			
	214	145	67.8%	145	15.6 y	7.8 y	8.0y	6.0–9.1 y	–
Sex									0.201
Male	166	102	61.4%	102	15.6 y	7.3 y	8.1y	6.3–9.6 y	
Female	48	43	89.6%	43	15.4 y	11.2 y	7.0 y	1.2–11.8 y	
Age group									<0.001
<50 years	35	16	45.7%	16	33.0 y	11.8 y	21.6 y	17.2–27.3 y	
50–59 years	52	24	46.1%	24	24.4 y	6.5 y	18.2 y	12.2–21.6 y	
60–69 years	65	45	69.2%	45	16.5 y	9.8 y	6.9 y	3.0–9.3 y	
70–85 years	62	60	96.8%	60	10.7 y	6.0 y	4.3 y	1.7–6.6 y	
Location									0.287
GAC	102	87	85.3%	87	15.5 y	9.8 y	7.4 y	4.3–8.8 y	
EAC	112	58	51.8%	58	15.7 y	6.4 y	8.2 y	5.0–11.3 y	
pT‐category									0.273
pT1a	90	56	62.2%	56	15.5 y	8.5 y	6.9 y	4.2–8.5y	
pT1b	124	89	71.8%	89	15.6 y	7.1 y	8.8 y	6.3–10.4y	
pN‐category									<0.005
pN0	181	118	65.2%	118	15.2 y	8.4 y	7.1 y	4.4–8.5y	
pN+	33	27	81.8%	27	16.5 y	3.0 y	11.5 y	6.6–11.4y	

Abbreviations: CI, confidence interval; EAC, esophageal adenocarcinoma; EYL, expected years of life adjusted for gender and age at time of surgical therapy; GAC, gastric adenocarcinoma; OYS, observed years of survival after time of surgical therapy.

**TABLE 5:** Comparison of tumor stages between males and females, for different age groups and between gastric (GAC) and esophageal (EAC) cancer for patients who died during follow‐up.

Factor	Patients died during follow‐up		pT‐category				Sign. *p*	pN‐category				Sign. *p*
	N	%	pT1a		pT1b			pN0		pN+		
			n	%	n	%		n	%	n	%	
	145	100%	56	39%	89	61%	–	118	81%	27	19%	–
Sex							0.080					0.115
Male	166	78%	42	41%	60	59%		80	78%	22	22%	
Female	48	22%	14	33%	29	67%		38	88%	5	12%	
Age group							0.815					0.023
<50 years	16	11%	5	31%	11	69%		9	56%	7	44%	
50–59 years	24	17%	8	33%	16	67%	*p*‐trend	18	75%	6	25%	*p*‐trend
60–69 years	45	31%	19	42%	26	58%	0.452	40	89%	5	11%	0.0131
70–85 years	60	41%	24	40%	36	60%		51	85%	9	15%	
Location							0.579					0.728
GAC	87	60%	32	37%	55	63%		70	80%	17	20%	
EAC	58	40%	24	41%	34	59%		48	83%	10	17%	

**FIGURE 2:**
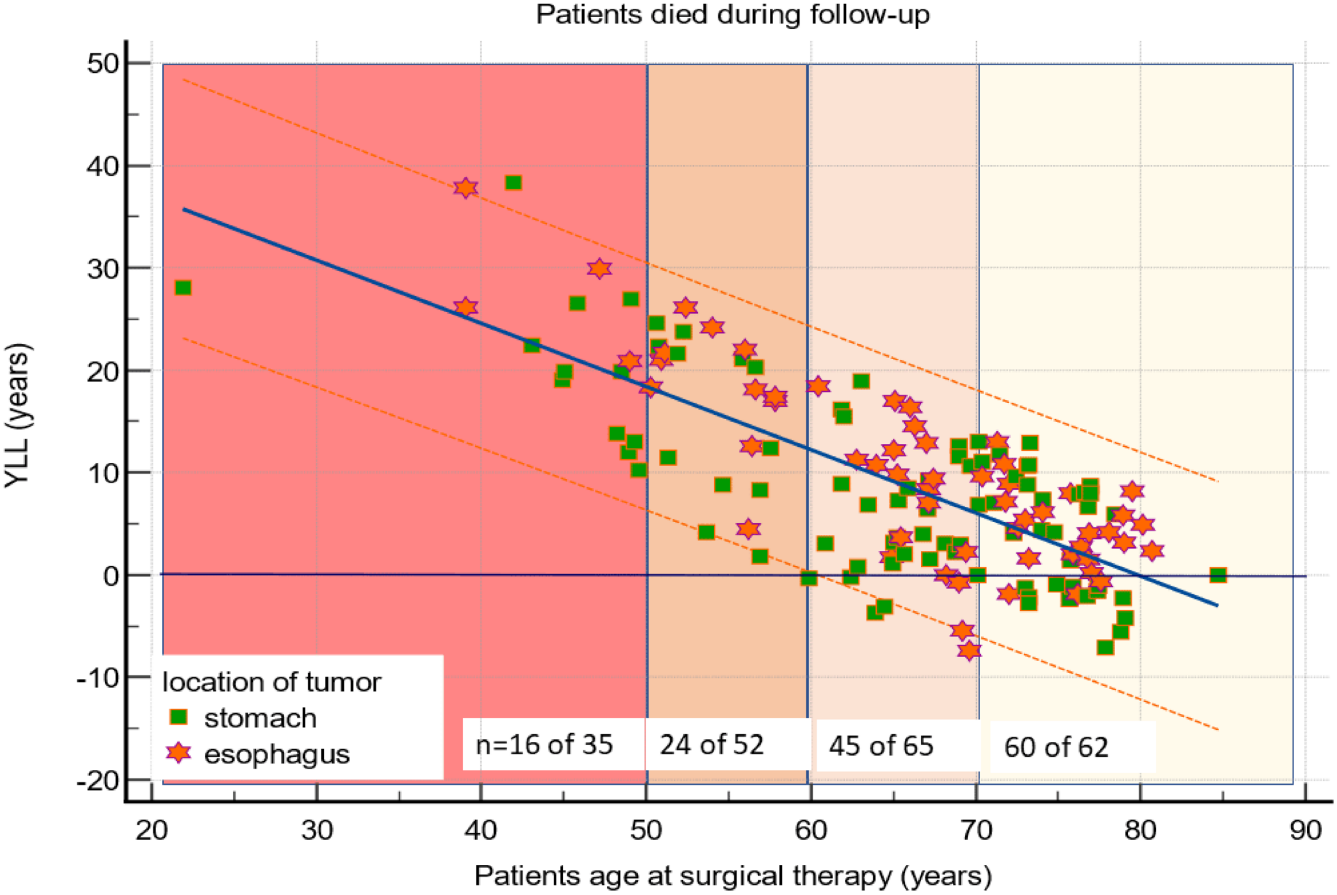
Regression line and the 95% prediction lines for the number of Years of Life Lost (YLL) according to the age of each patient at surgical therapy for 145 patients with early adenocarcinoma of the esophagus (EAC) or stomach (GAC) who died over the follow‐up period [YLL = 49.3 years − (0.62 × age of the patient at surgery) years].

Univariate analysis showed that age and the existence of LNM significantly influence the number of YLL. The median YLL was approximately 20 years for patients under 60 and approximately 5 years for the older group (Table 4 and Figure 1). For patients with early GAC, the median YLL was 7.4 years, and for those with early EAC, it was 8.2 years (Table 4 and Figure 1B). Figure 2 shows the linear regression and its 95% prediction lines for the number of YLL as a function of patients' age at the time of surgical therapy, YLL = 49.3y −0.62 × age of the patient at surgical therapy in years. The relevance of this function is that older patients lose less years of their life expectancy than younger one, with each year of increasing age, the number of YLL will be reduced by 0.62 years. Results were similar when analyzing the YLL for patients with EAC or GAC (Table 4 and Figure 2).

Forty patients younger than 60 years (46%) died during the follow‐up. Only one patient – 59.9 years old at the time of surgery – reached the expected time. In contrast, the observed survival was equal or longer than the expected survival in 25% in the group of patients ≥60 years.

**FIGURE 3:**
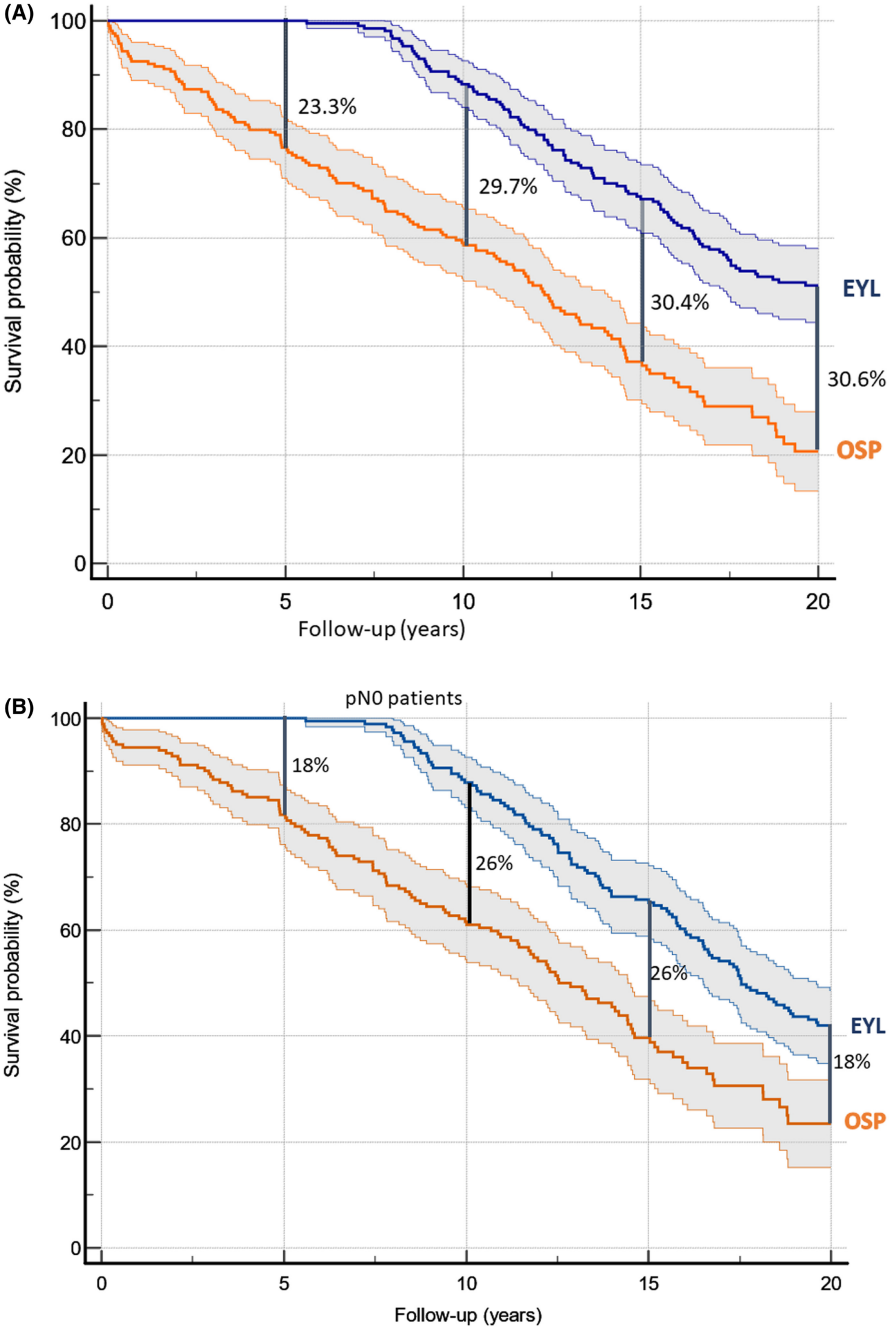
(A) Kaplan–Meier survival curves with 95% confidence interval for 214 patients with early gastric or esophageal adenocarcinomas comparing the expected survival with the observed survival probability. (B) Kaplan–Meier survival curves with 95% confidence interval for 181 patients with early gastric or esophageal adenocarcinomas without lymph node metastasis (pN0‐patients) comparing the expected survival with the observed survival probability. EYL, expected years of life adjusted for gender and age at time of surgical therapy; OSP, observed years of survival probability after time of surgical therapy.

We used the Kaplan–Meier survival calculation to estimate the survival difference between the normal population and all patients with EAC or GAC. For the expected years of life (EYL), the median was 17.8 (95%CI = 16.5–19.6) years, and the median of the observed survival probability (OSP) was 12.3 (95%CI = 10.9–14.0) years (*p* < 0.0001). Figure 3A shows that at 5, 10, 15, and 20 years, the observed OSP was 23.3%, 29.7%, 30.4%, and 30.6% lower than the expected survival probability.

Analyzing only the group of patients without lymph node metastasis, the results were similar with a median EYL of 17.5 (95%CI = 16.5–19.4) years and a median OSP of 12.5 (95%CI = 11.4–14.4) years (Figure 3B).

## 4 DISCUSSION

The results of this present study suggest that the burden of GAC or EAC diagnosis upon life expectancy is not absolutely dependent on the tumor biology alone. We analyzed data from patients with surgically treated early carcinoma in whom the tumor and the regional lymph nodes were completely resected. Thus, we hypothesized that the measured survival should be similar to the life expectancy of normal population given that we were studying the best prognostic group of patients with EAC and GAC (early stage). The short‐term data confirmed the expected good prognosis with a 5‐year survival rate of 77%. These results are comparable to other published data in similar patients.^22^, ^23^, ^24^

The most prognostic factor associated with mortality in the first 5 years after surgical resection of early carcinoma of the upper gastrointestinal tract was the presence tumor‐infiltrated regional lymph nodes. The important result highlights the need for consideration of adjuvant therapy including immunotherapy in these patients with postoperative positive lymph node status.^25^ A further important outcome of the study showed early‐stage cancer patients aged under 60 years may lose up to 20 years of their expected lifetime. With increasing age at diagnosis, the lifetime loss is reduced to approximately 5 years. The results of this study regarding the high YLL for tumor patients with a relatively excellent prognosis are of great importance when counseling patients regarding prognosis after esophageal and gastric cancer treatment.

In Germany, the median age at diagnosis for all stages of GAC is 71 years for males and 76 years for females; for EAC, it is 68 years for males and 71 years for females.^26^ In our study, 25% of the patients in the age group ≥60 years had survived longer than the estimated survival probability. The clinical relevance that can be drawn from the present data is that older patients with a curatively treated early carcinoma of the gastroesophageal junction have the same chance of life expectancy as the normal population. In our study, only patients with a low or moderate risk of comorbidities were included to keep the surgical risk low. The influence of risk factors to the reduced life expectancy may be more relevant.

Our study cohort with early‐stage cancer was younger, with a median age of 63.4 years. One‐third of the patients with GAC and nearly half of patients with EAC were younger than 60 years with the greatest YLL risk. Nearly all non‐survivors in this group had shorter survival compared to the life expectancy of an age‐ and gender‐adapted person of the normal population. The reasons may be 1. the tumor itself, 2. pre‐existence of comorbidity, and 3. limitations due to the therapy like the surgical procedure.

### 4.1 Influence tumor stage

Nearly two‐thirds of our patients had submucosal cancer without significant differences between age groups. Younger patients had significantly more often lymph node metastasis in the group of died patients but not in the whole group of patients (Tables 2 and 5). In the multivariate analysis, the existence of lymph node metastasis significantly influenced the prognosis during the first 5 years after surgery but not during the further follow‐up. Consequently, we have analyzed the data of patients without lymph node metastasis and complete resection of the primary tumor and demonstrated a substantially reduced life expectancy compared to the general population (Figure 3B).

### 4.2 Pre‐existence of comorbidity

For patients older than 60 years, the coronary risk is one relevant influence factor to the premature mortality. In our subgroup analysis, the coronary risk increases with age to more than 60%. Further, about one‐third of the patients had a BMI >25. Especially in the youngest patient group, overweight was seen more often. The impact of a high BMI to the outcome of younger patients with a longer life expectancy is open.

Some authors have compared younger gastrointestinal tumor patients (mostly <50 years) with older patient groups according to clinical characteristics or outcomes.^27^, ^28^, ^29^, ^30^, ^31^, ^32^, ^33^, ^34^ These studies showed increasing incidence of EAC,^30^ more poorly differentiated tumors,^30^ and more advanced tumor stages^28^, ^30^ for younger patients. Codipilly et al.^28^ used data from the SEER‐database and concluded that while young‐onset EAC remains uncommon, it is associated with worse 5‐year EAC‐free survival compared with older cohorts driven by advanced stage at diagnosis. Studies of the group of younger patients with GAC showed decreasing incidence in Germany,^31^ more frequent poorly differentiated tumors,^32^, ^33^ with a resulting poorer prognosis.^34^

### 4.3 Limitations due to the surgical procedure

During the first 5 years, one in four patients of our study cohort died. During this period and later the patients had symptoms caused by the changed anatomy by surgical procedure. Van Ernig et al.^35^ studied the incidence of gastrointestinal symptoms in the first year after resection of esophageal or gastric cancer. The study population consisted of 961 and 259 patients who underwent an esophagectomy and gastrectomy, respectively. For both groups, the majority of gastrointestinal symptoms changed significantly over time. At 9–12 years after resection, one or more severe gastrointestinal symptoms were reported by 38.9% after esophagectomy and 33.7% after gastrectomy. Dirr et al.^36^ compared the symptoms of patients with cancer‐free survival after Ivor Lewis esophagectomy with healthy controls. Compared with controls, patients had worse outcomes for dysphagia, GERD, Dumping Syndrome, and HRQL. These symptoms may be accompanied with impaired nutritional status.^37^ Cheng et al.^38^ found that in the follow‐up until 15 years persistent poor health‐related quality of life symptoms after esophagectomy were lower in patients with adenocarcinoma compared to squamous cell carcinoma and in earlier stage tumors.

For patients 70 years or older, nearly one in two patients died during this time span. We can speculate that the combination of oncologic burden, age‐related comorbidities, and the stress of surgical therapy with prolonged recovery are important factors associated with earlier death in the older group. In the younger groups, the risk factors for these tumor entities may be especially relevant for mortality along with the influence of tumor burden and therapy upon immune function.

To date, only a few studies have discussed the life expectancy burden of tumor diagnosis. Lundberg et al. performed a population‐based study including all patients treated for esophageal cancer in Sweden and surviving 5 years.^39^ They found that esophageal cancer survivors had poorer survival than the corresponding background population, and the difference increased from 6 to 10 years after surgery. However, all tumor stages and both tumor histology types were included in their analysis. Participants with a history of EAC showed only a slightly decreased relative survival over follow‐up, with a reduction from 96.9% (95% CI 94.8–99.0%) in year 6 to 91.5% (95% CI 86.6–96.3%) in year 10. These data were comparable with our results. Figure 3A shows that over follow‐up from 5 to 10 years, the difference between expected and OYS rate increased by 6.4%. Pham et al.^40^ published data regarding premature mortality due to gastric cancer in Japan. They found that the average lifespan reduction changed for males from 21.9% in 1980 to 13.6% in 2015. Although Japanese patients with gastric cancer are typically diagnosed at an early tumor stage, the differing etiology for this tumor type make the results not reliably comparable.^41^

In this paper, we have focused upon the number of years of life loses as a consequence of dying early because of early carcinoma of the gastroesophageal junction. The concept of “the overall burden of disease” add to the number of YLL, the adjusted number of years of life a person lives with disability caused by this disease. According to the literature, it is well established that the health‐related quality of life (HRQoL) is substantially adversely affected by the panacea of different symptoms patients experience after esophagectomy or gastrectomy.^42^, ^43^ Thus, overall impact of surgical treatment in patients with early‐stage cancer will require further investigation with assessment of both YLL and HRQoL. Furthermore, future clinical trials seeking to evaluate the potential benefit of an organ presentation strategy in this cohort of patients with early‐stage disease should be designed with equal value to both YLL (survival) and HRQoL.

The strength of our study is the inclusion of patients from one high‐volume center for esophageal and gastric surgery with nearly complete follow‐up with a median follow‐up for living patients of 14 years. A weakness is that we could not evaluate the causes of death of all patients because these data were not available at the registration office. It would be of interest for further studies to determine whether similar results are obtained after endoscopic treatment of early esophageal or gastric adenocarcinoma.

## 5 CONCLUSION

The results of this study showed that patients with early‐stage AC of the stomach or esophagus had a distinctly worse life expectancy after curative surgical therapy than the corresponding general population in Germany. Younger patients in particular showed the greatest life expectancy burden due to these diseases. In contrast, older patients have the chance for a normal life expectancy. A careful follow‐up, taking in consideration symptoms resulting from the tumor disease, could reduce comorbidities. Early prevention of risk factors, including obesity, may reduce the number of new cases and relevant comorbidities and may be important for overall survival after radical surgical resection.

## AUTHOR CONTRIBUTIONS

**Elfriede Bollschweiler:** Conceptualization (lead); formal analysis (lead); writing – original draft (equal); writing – review and editing (equal). **Arnulf H. Hölscher:** Conceptualization (equal); project administration (equal); writing – review and editing (equal). **Sheraz R. Markar:** Methodology (equal); validation (equal); writing – review and editing (equal). **Hakan Alakus:** Data curation (equal); project administration (equal). **Uta Drebber:** Data curation (equal); formal analysis (equal); methodology (equal). **Stefan Paul Mönig:** Project administration (equal); supervision (equal). **Patrick Sven Plum:** Conceptualization (supporting); data curation (equal); project administration (supporting); validation (equal).

## CONFLICT OF INTEREST STATEMENT

All authors declare that there is no conflict of interest.

## ETHICS STATEMENT

The study was performed according to the local Research Ethics Guidelines and Helsinki‐Ethical Principles. An application for ethical approval was submitted to the local independent Ethics Committee of the University of Cologne (692732). Written informed consent for collection of patient data was obtained from all patients within this study prior to surgery.

## Data Availability

The data that support the findings of this study are available from the corresponding author upon reasonable request. For expected survival the following database were used: Genesis. Durchschnittliche Lebenserwartung in Deutschland. https://www‐genesis.destatis.de/genesis/online.
